# Noseleaf Dynamics during Pulse Emission in Horseshoe Bats

**DOI:** 10.1371/journal.pone.0034685

**Published:** 2012-05-04

**Authors:** Lin Feng, Li Gao, Hongwang Lu, Rolf Müller

**Affiliations:** 1 SDU-VT International Laboratory, School of Physics, Shandong University, Jinan, Shandong, China; 2 Department of Mechanical Engineering, Virginia Tech, Danville, Virginia, United States of America; University of Salamanca-Institute for Neuroscience of Castille and Leon and Medical School, Spain

## Abstract

Horseshoe bats emit their biosonar pulses nasally and diffract the outgoing ultrasonic waves by conspicuous structures that surrounded the nostrils. Here, we report quantitative experimental data on the motion of a prominent component of these structures, the anterior leaf, using synchronized laser Doppler vibrometry and acoustic recordings in the greater horseshoe bat (*Rhinolophus ferrumequinum*). The vibrometry data has demonstrated non-random motion patterns in the anterior leaf. In these patterns, the outer rim of the walls of the anterior leaf twitches forward and inwards to decrease the aperture of the noseleaf and increase the curvature of its surfaces. Noseleaf displacements were correlated with the emitted ultrasonic pulses. After their onset, the inward displacements increased monotonically towards their maximum value which was always reached within the duration of the biosonar pulse, typically towards its end. In other words, the anterior leaf’s surfaces were moving inwards during most of the pulse. Non-random motions were not present in all recorded pulse trains, but could apparently be switched on or off. Such switches happened between sequences of consecutive pulses but were never observed between individual pulses within a sequence. The amplitudes of the emitted biosonar pulse and accompanying noseleaf movement were not correlated in the analyzed data set. The measured velocities of the noseleaf surface were too small to induce Doppler shifts of a magnitude with a likely significance. However, the displacement amplitudes were significant in comparison with the overall size of the anterior leaf and the sound wavelengths. These results indicate the possibility that horseshoe bats use dynamic sensing principles on the emission side of their biosonar system. Given the already available evidence that such mechanisms exist for biosonar reception, it may be hypothesized that time-variant mechanisms play a pervasive role in the biosonar sensing of horseshoe bats.

## Introduction

Microbats have evolved active biosonar systems that analyze the echoes created by the emission and reflection of ultrasonic pulses. In these sensory systems, the incoming ultrasonic waves of the echoes are always transformed through diffraction by the shapes of the outer ears [1], [2]. These transformations can be functionally relevant because they could be used to enhance the encoding of sensory information [3], [4].

In addition to the diffraction of the incoming waves, a significant number of microbat species have evolved specialized structures that diffract the outgoing waves upon emission. These emission-side structures are particularly common and conspicuous in bat species that emit their ultrasonic pulses through the nostrils, such as the Old-World horseshoe bats (*Rhinolophidae*) and the New-World leaf-nosed bats (*Phyllostomidae*). In these bat groups, the baffles that diffract the outgoing waves are referred to as noseleaves.

As for the outer ears, the geometry of the noseleaves determines the outcomes of the diffraction process together with the direction of the outgoing or incident sound as well as its frequency content [2], [5]–[7]. When operating in the acoustic far-field, the resulting system properties are commonly described by beampatterns (gain as a function of direction for fixed frequencies) or head-related transfer functions (HRTFs, gain as a function of frequency for fixed directions). Both descriptions can be applied to emission as well as to the reception side of the biosonar system. Beampatterns as well as HRTFs are frequency-domain characterizations that assume a time-invariant system. As a consequence, the techniques that are currently in use to characterize the acoustic properties of noseleaves and pinnae in bats are limited to representing time-invariant features.

Horseshoe bats have evolved a highly advanced biosonar system [8] that includes adaptivity on multiple levels. Of particular note is the dynamic control that these animals exert over the carrier frequency of their biosonar pulses in order to compensate for Doppler shifts [9]. This raises the question whether such time-variant adaptivity also exists for the baffle shapes that diffract outgoing and incoming ultrasound. On the reception side, it has been demonstrated already that the geometry of the outer ear surfaces can be time-variant: Horseshoe bats have been found to change the shapes of their outer ears to an extent that could alter their acoustic function significantly [10]. These non-rigid ear deformations occurred over sub-second time intervals (e.g., complete deformations within 100 milliseconds) that were not too far away from the pulse durations (around 50 milliseconds under laboratory conditions) [10].

On the emission side, shape changes of diffracting baffles have yet to be demonstrated by experimental data. Noseleaf vibrations have been suggested as a mechanism of noseleaf function as an ultrasonic transducer [11]. This hypothesis would require noseleaf vibrations in the same frequency band as the ultrasonic emissions of the respective bat species.

In horseshoe bats, casual observation of animals emitting biosonar pulses indicates that at least one portion of the noseleaf can be set in motion during biosonar behaviors. This part is called the horseshoe (anterior leaf) [12]. The horseshoe forms a concave, incomplete baffle – not unlike an acoustic horn – that surrounds the nostrils rostrally and laterally.

The aim of the research presented here was to obtain experimental data on the motion of the anterior leaf in horseshoe bats during pulse emission. These experiments were intended to establish the motion of the anterior noseleaf through objective and quantitative data. Furthermore, the experimental data was intended to characterize the temporal patterns of these motions and in particular how they relate to pulse emission.

Obtaining such data is a necessary basis for future investigations of the function that these motions could potentially have. For example, if motions were restricted to the gaps between biosonar pulses, it would be exceedingly difficult to argue that they could have any relevant effects on pulse emission. The goal of the present research was hence limited to these basics that need to be clarified before the design of any further experiments.

## Results

**Figure 1:**
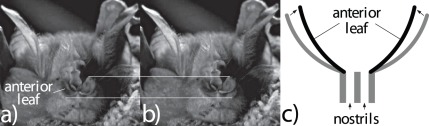
Deformation pattern of the anterior leaf. a,b) two subsequent frames (spacing 1/30 s) from a video recording of the anterior leaf vibration. The horizontal white lines provide a reference for comparing the positions of the anterior rim of the leaf. c) schematic cross-section through the deforming anterior leaf, gray: cross-section of the anterior leaf at rest, black: cross-section of the anterior leaf when twitching forward.

The deformations of the anterior portion (horseshoe) of the bats’ noseleaf that were observed during the emission of biosonar pulse trains followed a stereotypical spatial pattern (s. Figure 1). During qualitative (video) observations of these patterns, the outer rim of the anterior leaf could be seen to move away from the head and inwards whereas the rim of the anterior leaf around the nostrils stayed approximately in place. This motion hence resulted in a non-rigid deformation of the anterior leaf that narrowed its opening aperture and increased the curvature of the leaf surfaces.

**Figure 2:**
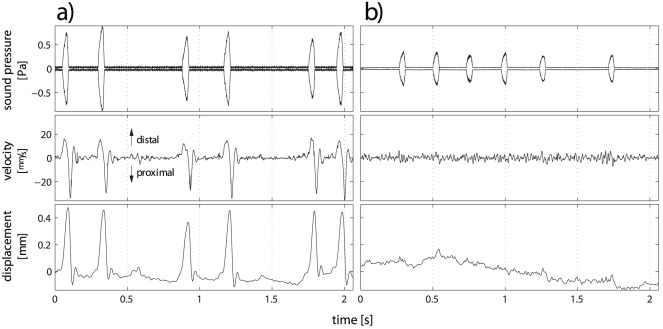
Examples of biosonar pulse sequences with synchronized measurements of the velocity of the anterior rim of the noseleaf in the proximal-distal direction. a) example sequence with non-random motions of the anterior leaf. b) example sequence without non-random motions. For each example, the top graph shows the envelope of the sound pressure amplitude, the center graph the velocity, and the bottom graph the displacement amplitude of the anterior rim of the noseleaf in the proximal-distal direction. Positive velocities and displacements correspond to motions in the distal direction (i.e., away from the head).

Quantitative measurements of the movement of individual points on the anterior leaf with laser Doppler vibrometry showed that the anterior leaf motions followed a temporal pattern that consisted of individual, biphasic velocity pulses: In each of these pulses, a first phase with velocities in the distal direction (i.e., away from the head) was followed by a second phase with velocities in the opposite direction (s. Figure 2a, center graph). Typically, the peak velocities in the phase of distal motion were smaller than those in the phase of proximal motion, but the distal motions extended over a slightly larger period of time. As a result, the anterior leaf returned approximately to its original position after each biphasic velocity pulse. The maximum displacements measured were as high as 0.75 mm with a mean of 0.35 mm and a standard deviation of 0.21 mm (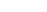, s. Figure 2a, bottom graph). After the large velocity excursions of the first two phases, the anterior leaf can go through a short “ringing phase” (s. Figure 2a) before it settles into its resting position. However, the displacement amplitudes during this ringing phase were always very small compared to those associated with the initial two phases and not always distinct from the noise-level of the respective recordings.

Not all pulse sequences emitted by the bats in the experiments were accompanied by distinctive velocity and displacement pulses. If a recording lacked distinct velocity and displacement pulses (s. Figure 2b for an example), it did not show any other discernible non-random patterns either. As a consequence, all recordings fell into one of two categories, showing either distinctive velocity pulses or only random noise. The data set analyzed here contained six biosonar pulse sequences with a non-random velocity pattern and a total of 52 pulses. In addition, seven sequences with only random noise in the velocity signal and 48 biosonar pulses were analyzed. Subsequent biosonar pulses within a continuous train where always homogeneous in terms of the presence or absence of non-random motions of the anterior leaf. The bats were never found to alter between pulses with and without accompanying non-random motion patterns from one individual pulse to the next.

**Figure 3:**
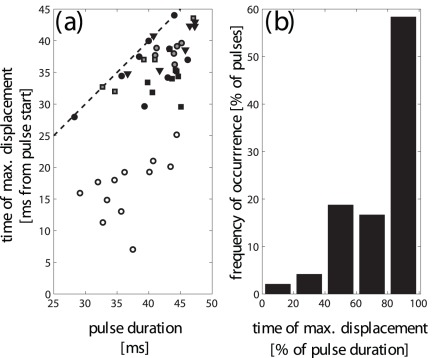
Distribution of the position of the maximum displacement amplitudes within the pulse duration. a) position of the maximum displacement within the pulse as a function of pulse length, b) histogram of the position of the maximum displacement within each pulse in percent of pulse duration. In a), different marker shapes denote different individuals and different gray levels of the marker faces (including black and white) signify different pulse sequences. The dashed line in a) marks the pulse duration, i.e., all the points falling below this line indicates that the maximum displacement never occurred after the end of the pulse.

Synchronized recordings of anterior leaf velocities and sonar pulses showed close temporal correlations between the emitted pulses and the motions of the anterior leaf (s. Figure 2a). The duration of the pulses analyzed ranged from around 37 ms to about 42 ms. Each biosonar pulse in sequences with large anterior leaf motions was accompanied by a biphasic velocity pulse that caused a single distal displacement of the anterior leaf. In all analyzed pulses, the time of occurrence of the anterior leaf’s maximum displacement fell within the duration of the pulse (s. Figure 3). In the majority of cases, the maximum displacement occurred towards the end of the pulse (s. Figure 3). In about 70 percent of the pulses analyzed (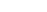), the maximum displacement fell within the terminal third of the pulse duration. This means that for most of the duration of a typical pulse, the surfaces of the anterior leaf were moving in the distal direction.

**Figure 4:**
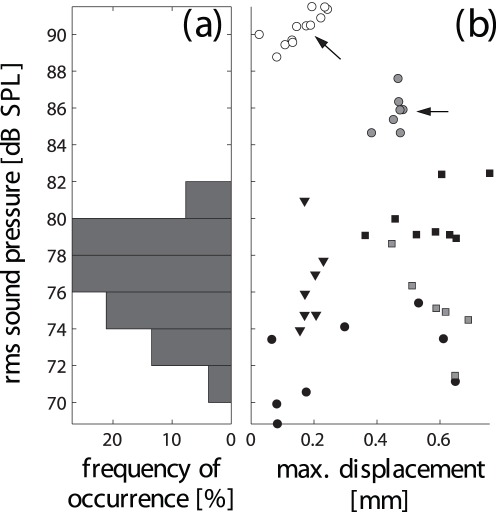
Joint distribution of the recorded pulse data set over maximum motion amplitudes and recorded sound pressure levels. a) distribution of sound pressure amplitude values for pulses without detectable motions of the anterior leaf, b) joint distribution of the analyzed pulses with respect to displacement and sound pressure level. In b), different marker shapes denote different individuals and different gray levels of the marker faces signify different pulse sequences. The pulse data and encoding of the individuals and sequences is the same as in Figure 3. Arrows mark the two pulse sequences that fell outside the range of sound pressure amplitude covered by the pulses not accompanied with detectable anterior leaf motions.

Within the set of recorded pulses that were accompanied by non-random noseleaf motions, the magnitude of the anterior leaf displacements was not found to correlate with the rms sound pressure level of the emitted pulses (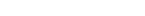, s. Figure 4b). Likewise, most of the rms sound pressure amplitudes recorded for pulses accompanied by large displacements fell within the range of the amplitudes recorded for sequences without non-random displacements (71 to 82 dB SPL, s. Figure 4a). Only two of the recorded sequences (from the same individual) exceeded this range (s. Figure 4a and b) with rms sound pressure levels of about 86 and 90 dB SPL (rounded sequence averages).

## Discussion

The data obtained in the current study has demonstrated a non-random pattern of motions occurring in the anterior portion of the noseleaf in horseshoe bats. When present, these motions were found to be correlated with the emission of the biosonar pulses and always occurred within the duration of the pulse.

While the data obtained thus far clearly documents this effect, it is not well suited to shed light on its causes or potential functions. Hence, it is still possible at the present stage of the research that the observed noseleaf motions could be a side effect rather than a functional feature in its own right. An example explanation of the motion of the noseleaf as a side effect could be that it is caused by pushing strong air flows through the vocal tract with each pulse. However, the lack of a correlation between the noseleaf displacement amplitudes and the sound pressure levels – even over a level range of more than 20 dB – may be seen as not in favor of hypotheses of this kind.

A more weighty argument against the noseleaf motion being such a side effect is that the bats appear to have control over the motion and seem to be able to turn it off entirely (s. Figure 2b). At the same time, there seems to be no systematic difference between the pulse amplitudes (apart from perhaps the two recorded outlier sequences, s. Figure 4). The fact that the bats can produce a sequence of pulses with equal amplitudes with as well as without accompanying noseleaf motions contradicts the hypothesis that the motions are “automatic” byproducts of pulse emission. Qualitative observations made during the experiments indicates that noseleaf motions seems to be often triggered by novel targets.

The velocity and displacement signals that accompanied each biosonar pulse occupied a much lower frequency band than the ultrasonic pulses themselves. Vibrations in the same frequency band as the biosonar pulses as hypothesized in [11] were not observed in this study, although the specifications of the experimental setup (e.g., vibrometer bandwidth and sampling frequency) would have been sufficient to detect them. Hence, the current study provides no evidence to support the hypothesis that noseleaves in bats act as a vibrating ultrasonic transducers. At least for the horseshoe bat species and the experimental conditions studied here, this hypothesis can thus be ruled out.

Compared to the speed of sound, the noseleaf velocities that were observed in the present work were very small, i.e., noseleaf surface velocities of less than 3 cm/s (s. Figure 2a) appear to be negligible compared with a sound speed that exceeds 300 m/s. This difference of four orders of magnitudes could only cause non-linear signal transformation by virtue of Doppler effects that would be in the range of a few Hertz. Such small frequency effects are well below the frequency resolutions of the horseshoe bats that are manifest in filter quality or the precision of Doppler shift compensation [13]. This makes it unlikely that a possible acoustic function of the noseleaf motions could act through Doppler effects induced by the velocity of the anterior leaf. However, it is difficult to rule out any special circumstances in the high-amplitude – and hence perhaps non-linear – acoustic near-fields surrounding the anterior leaf that could still make such small effects relevant.

In contrast to the velocities, the change in the geometry of the anterior leaf is of a magnitude that could readily be relevant to the acoustic function of the anterior leaf. In many cases (11 out of 48 pulses analyzed), the estimated displacement were found to exceed one eighth of the wavelength associated with the constant frequency portion of the animals’ biosonar pulses. Since this displacement takes place on both sides of the anterior leaf, it could result in an overall effect on the noseleaf aperture that could amount to as much as a quarter wavelength in magnitude. At such size scales relative to the wavelength, the changes in geometry could be hypothesized to affect the radiation pattern of the bats’ biosonar emissions. By comparison, the breadth (diameter) of the entire anterior leaf in the greater horseshoe bat falls between 6.5 and 9.9 mm [14], which is equivalent to about 1.5 to 2.5 wavelengths of the constant frequency portion of the pulses.

If the noseleaf deformations reported here were to have acoustic effects of functional relevance, the biosonar system of horseshoe bats would be capable of exploiting time-variant effects on the emission as well as on the reception side [10]. The presence of such effects on both sides of this active sensing system could then be taken as an indication of their fundamental importance. In this scenario, the currently used time-invariant characterization methods would miss critical functional aspects entirely. In this case, analysis techniques devised to look specifically at the dynamic nature of the system would be needed. If successful, such investigations could open up the opportunity to discover novel, time-variant principles of sensing that could be of interest to biology as well as to engineering.

## Materials and Methods

A total of three adult greater horseshoe bats (*Rhinolophus ferrumequinum*), one male and two females, were obtained from caves in the vicinity of Jinan, Shandong province. The bats were housed in the laboratory for short time periods prior to recording of the noseleaf deformations and fed on a diet of mealworms and water (available ad libitum). The animals were not deprived of food to influence their behavior in the experiments.

**Figure 5:**
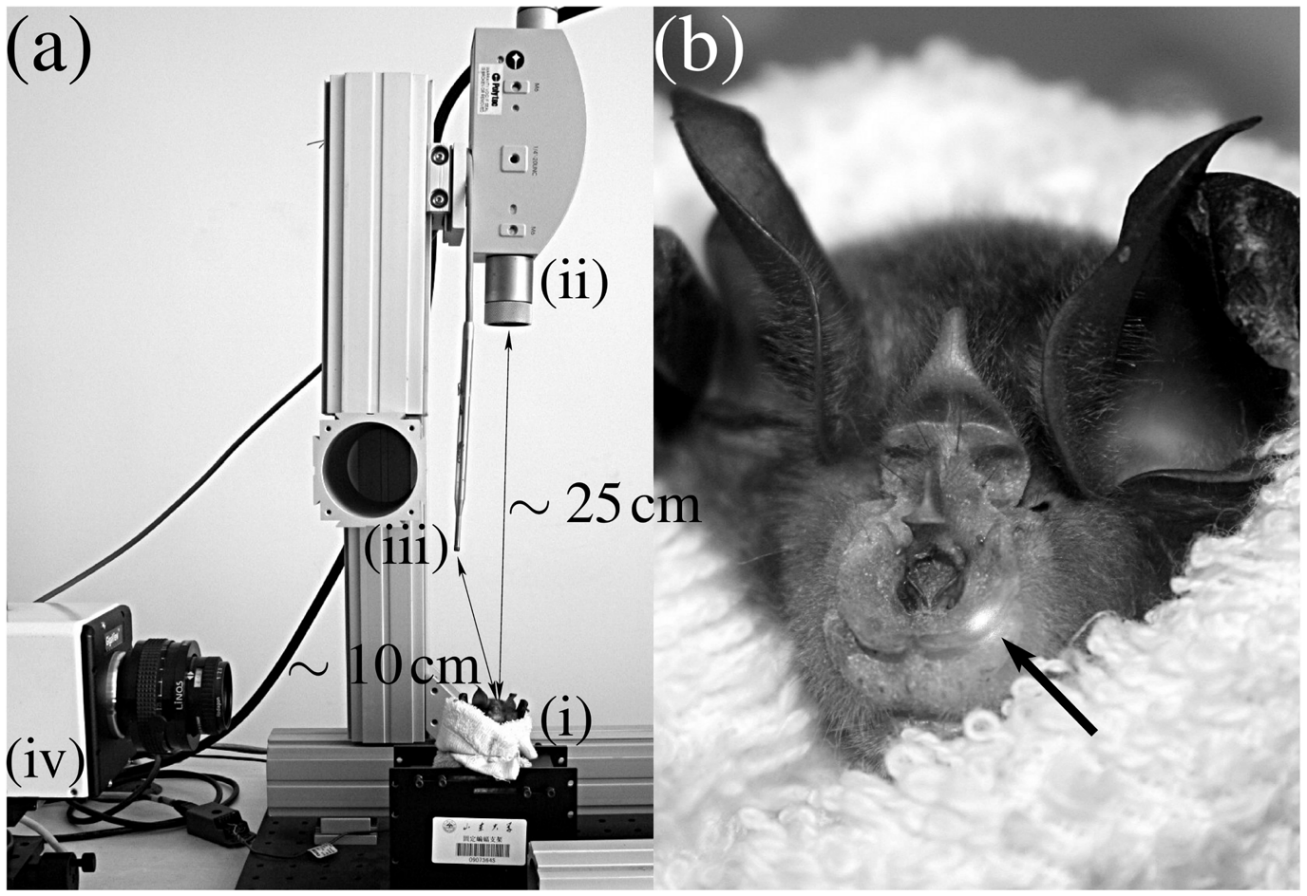
Experimental setup. a) overall layout of the setup: (i) bat, (ii) sensor head of the laser Doppler vibrometer, (iii) microphone, (iv) high-speed video camera, b) experimental subject with laser spotlight (arrow) on the anterior leaf. During experiments, the orientation of the experimental subjects was continuously adjusted by hand.

During laser Doppler vibrometry measurements, the wings and the body of the bats were confined in a small towel that was loosely wrapped around the animals. The animals were accustomed to being wrapped in the towel by hand-feeding them in this situation. The towel covered the animals’ neck (s. Figure 5b) which was found to greatly reduce head movements. However, no change in the noseleaf motions was evident from qualitative observation. During laser Doppler measurements, the animals were held in hand and oriented so that the laser beam was impinging approximately normal to the anterior leaves of their noseleaves. At the same time, the animals’ eyes were kept away from the laser’s path. The arrangement shown in Figure 5a does not include the continuous manual adjustment of the animals orientation. The bats were enticed to emit sequences of biosonar pulses by presenting them with a variety of new, moving targets. The animals were not trained to perform any specific sonar task for the current study.

A laser Doppler vibrometer (model No. OFV-2500-2 with sensor head OFV-534, Polytec GmbH Waldbronn, part ii in Figure 5a) was used to measure the velocity of the anterior portion of the noseleaf from a distance of about 25 cm. The displacement amplitudes were estimated through numerical integration of the measured velocity time series.

The biosonar pulses were recorded with an 1/8′′ pressure-field microphone (Brüel & Kjær, model 4138-A-015 with input/output module 3110 and Pulse multi-analyzer system 3560C, part iii in Figure 5a). The microphone capsule was placed at a distance of approximately 10 cm from the bat’s head.

The analog output signals of the laser Doppler vibrometer and the sound measurement amplifier were digitized via two synchronized input channels of a data acquisition system (National Instruments RIO platform, model NI R7852). The sampling rate was set to 500 kHz for each channel. The highest frequency in the input signals was found in the biosonar pulses. In these signals, the constant frequency components of the second harmonic that with the most signal energy were found to be around 78 kHz.

A high-speed video camera (Gigaview by Southern Vision Systems, Inc., part iv in Figure 5a) was used to monitor the experimental process and ensure that the recorded velocities corresponded to motion of the anterior leaf relative to the head and not to motions of the entire animal or its head. The high-speed video recordings were synchronized with the ultrasound acquisition and the laser Doppler vibrometry by a common trigger signal, but were analyzed only in a qualitative fashion that was sufficient to establish the origin of the velocity signal from the motion of the anterior leaf: Looking at the video, it could be verified that the anterior leaf was in motion relative to the head during the recording. Furthermore, the video recordings allowed a verification that properties of the recorded velocity signal such as repetition rate and displacement amplitude approximately matched coarse estimates of the same parameters obtained from the video. A significant influence of head motions on the recordin’gs could be excluded by virtue of the video recordings, since the laser beam was approximately parallel to the image plane of the high-speed video camera (s. Figure 5a), so any significant head motion affecting the vibrometry results would have been detectable by comparing subsequent video frames. Recordings were triggered manually and each recording lasted for a period of 3 seconds.

For the results discussed here, a total of 13 pulse sequences were analyzed. In six of these sequences, non-random patterns of anterior velocities/displacements were clearly detectable, in the other seven, such patterns were completely absent. From these sequences, a total of 100 pulses were analyzed, 52 with non-random velocity patterns and 48 without. Non-random velocity patterns were defined as having amplitudes and temporal shapes that clearly deviated from the observed noise base line. This qualitative decision could be made readily for all analyzed sequences, i.e., each analyzed sequence either contained clearly visible biphasic velocity pulses with amplitudes well above the noise level or no non-random components at all.

**Figure 6:**
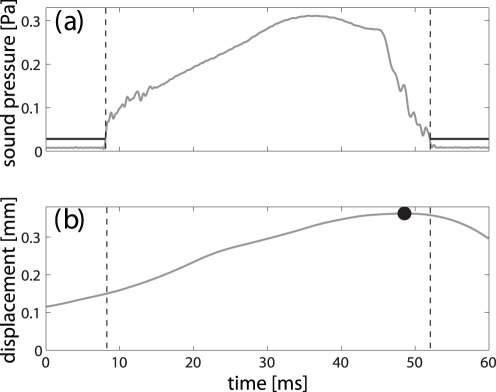
Estimation of the temporal position of the maximum displacement within the pulse. a) sound-pressure envelope with amplitude threshold used to determine the pulse time window, b) position of the maximum displacement (circle) within the pulse time window.

In order to assess the relative timing of the biosonar pulses and the motions of the anterior leaf, the temporal position of the maximum displacement of the anterior leaf was determined relative to the pulse duration. The duration of the biosonar pulses was determined by thresholding the envelope of the analytic signal (s. Figure 6) with a threshold set at five times the root mean square (rms) amplitude of the noise in each sound recording. Since the displacement curves were found to be generally smooth and with a clearly distinguished maximum, the location of the displacement maximum was determined by finding the maximum value of the time series. The same thresholding approach to pulse detection was also used for computing the root-mean-square (rms) sound pressure amplitudes of the biosonar pulses (s. Figure 4).

### Ethics Statement

All animal work has been conducted in accordance with the relevant guidelines and regulations of the People’s Republic of China. Experiments were carried out under institutional permit number 2011-1487 from the Shandong University School of Physics Animal Care Committee that specifically approved this study. The animals were carefully accustomed to being handled and their orientation and head movements in the experimental setup were carefully controlled to avoid the laser beam shining into the eyes of the animals.
